# The Role of the Gut Microbiota in Mental Health and Cognitive Function in Patients with Coronary Atherosclerosis

**DOI:** 10.3390/nu17142311

**Published:** 2025-07-14

**Authors:** Paulina Helisz, Karolina Krupa-Kotara, Weronika Gwioździk, Joanna Głogowska-Ligus

**Affiliations:** 1 Department of Epidemiology, Faculty of Public Health in Bytom, Medical University of Silesia in Katowice, 41-902 Bytom, Poland; w.gwiozdzik@wp.pl (W.G.); jglogowska@sum.edu.pl (J.G.-L.); 2 Doctoral School of the Medical University of Silesia in Katowice, Faculty of Public Health in Bytom, Medical University of Silesia in Katowice, ul. Piekarska 18, 41-902 Bytom, Poland; 3 Cracow Higher School of Health Promotion Having Its Registered Office in Cracow, Health Promotion Faculty, 31-158 Cracow, Poland

**Keywords:** gut microbiota, coronary artery atherosclerosis, TMAO, cognitive impairment

## Abstract

The gut microbiota plays an important role in maintaining the body’s homeostasis, and its disruption has been linked to the pathogenesis of coronary atherosclerosis and cognitive decline. This review attempted to assess whether the composition of the gut microbiota differs significantly according to the severity of coronary atherosclerosis and whether the presence of specific cytokines and inflammatory markers in the microbiota of patients with atherosclerosis may correlate with cognitive impairment. In addition, it considered whether increased dietary fiber intake may contribute to lower levels of inflammatory markers compared to a low-fiber diet. This review included publications from 2015 to 2024, searched in the PubMed and Scopus databases. Only studies meeting the quality criteria were included. The pooled data indicate that intestinal dysbiosis can lead to increased intestinal barrier permeability and lipopolysaccharide (LPS) translocation, which promotes chronic inflammation. This process plays an important role in both atherosclerosis and neurodegeneration. In addition, some studies indicate a beneficial effect of dietary fiber in reducing inflammatory markers. The conclusions of this review highlight the need for further, well-designed studies to identify the causal relationship between the microbiota, its metabolites, atherosclerosis, and cognitive deficits, which may provide the basis for new therapeutic strategies.

## 1. Introduction

The term “microbiota” refers to the community of microorganisms (bacteria, fungi, and archaea) that colonize the human body, both on the surface of the body (e.g., skin microbiota) and internally (e.g., genitourinary microbiota). The gut microbiota, which is the most extensively studied microbial community, plays a key role in many physiological processes in the human body. Key functions include synthesis of essential vitamins (mainly B vitamins) and short-chain fatty acids (SCFAs), support of digestive processes, and modulation of the immune system [1,2,3].

A proper balance of bacterial strains is a key element in maintaining the body’s homeostasis. Any imbalance in the composition of microorganisms residing in the large intestine, or dysbiosis, can directly contribute to the development of diseases of various origins, including cardiovascular disease. Thus, any interaction between the host and the microbial community plays a fundamental role in maintaining health [1,2,3,4]. The diversity of the gut microbiota is influenced by many factors, mainly lifestyle, which includes, among others, diet and physical activity. Chronic stress, which is associated with, among others, hypercortisolemia, significantly implicates the composition of the gut microbiota, as well as contributing to the development of cardiovascular disease, including coronary artery atherosclerosis and cognitive impairment [3,4,5,6].

The present study aimed to investigate the hypothesis regarding the role of gut microbiota in mental health and cognitive function in patients with coronary artery atherosclerosis to better understand the potential links between the gut system and brain health in the context of cardiovascular disease. Coronary artery atherosclerosis is a known risk factor for mental disorders and cognitive decline, and a growing body of research suggests that gut microbiota may play a key role in this. To achieve the stated aim, the following research questions were posed:

Q1: Does the composition of the gut microbiota differ significantly according to the severity of coronary artery atherosclerosis?

Q2: Does the gut microbiota of patients with coronary artery atherosclerosis contain specific cytokines or inflammatory markers that are associated with cognitive impairment?

Q3: Can increasing dietary fiber intake reduce inflammatory markers in people with coronary artery atherosclerosis compared to a low-fiber diet?

## 2. Materials and Methods

### 2.1. Methodology Background

Studies suggest that microbiota may influence the gut–brain axis by modulating inflammation, neurotransmitter metabolism, and biological barriers such as the blood–brain barrier. Relationships between gut microbiota and cognitive function may point to new directions for the treatment and prevention of cognitive disorders accompanying coronary atherosclerosis. A review of the literature in this area aims to identify mechanisms through which the microbiota can potentially influence the gut–brain axis, including inflammation, neurotransmitter metabolism, and the blood–brain barrier.

### 2.2. Review Procedure and Search Strategy

The following paper has been edited in accordance with the established good practices commonly employed in works of this nature. The authors of this paper commenced by delineating the research field. To this end, they conducted a search of the PubMed database, whereby they identified scientific publications that were pertinent to the subject matter under consideration. The literature items were then subjected to research by the authors of this paper and a duly qualified library employee, utilizing pertinent keywords with Boolean operators and their combinations and configurations, including “gut microbiota”, “coronary atherosclerosis”, and “cognitive dysfunction”. This was conducted using the methodological tool in the form of the PubMed database.

### 2.3. Source Selection

**Figure 1:**
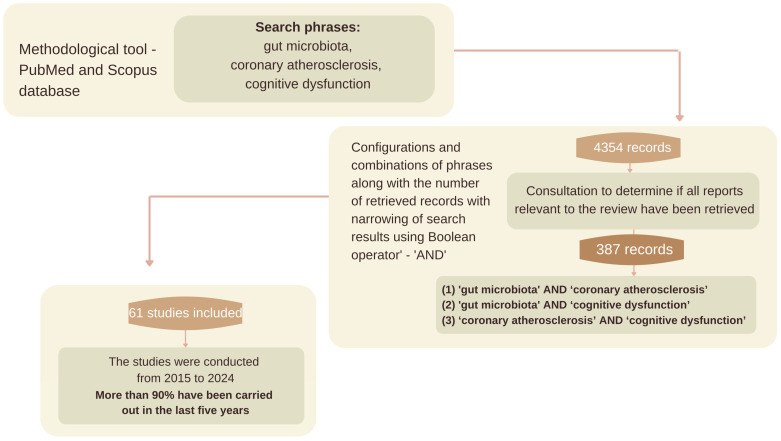
Methodological scheme.

A comprehensive literature search yielded a substantial number of records, from which 4354 sources were selected for their direct relevance to the topic of this paper. The selection process involved further refinement based on bibliometric impact factors, resulting in a final literature review comprising 387 sources, primarily representing scientific output from the recent five-year period (Figure 1).

The reliability, accuracy, and relevance of the work were evaluated using the GRADE (Grading of Recommendations, Assessment, Development, and Evaluation) system. This system was developed with the objective of eliminating confusion arising from the use of different evaluation methods. Consequently, an overview paper was prepared based on 61 scientific sources from 2015 to 2024. Particular attention was paid to the most recent scientific studies from the last five years, which account for over 90% of the cited sources. The exclusion criterion was scientific articles published before 2015 in order to keep the data analyzed up to date and to include only the most recent reports in light of the rapidly evolving knowledge of the gut microbiota.

## 3. Gut Microbiota

The human gut microbiota is already formed during fetal development. In the first days of a newborn’s life, the large intestine is mainly populated by bacterial strains such as Escherichia coli and Enterococcus faecalis. These microorganisms create suitable anaerobic conditions that allow for subsequent bacteria such as Bacteroides, Bifidobacterium, or Clostridium to colonize. Many factors influence the microbial composition of the gastrointestinal tract, shaping the diversity of the gut microbiota at different stages of human life. One key determinant is the prenatal stage, where factors such as gestational age or maternal diet, for example, play an important role in the colonization of the child’s gut microbiota. In addition, equally important aspects influencing the modulation of the human gut microbiota are the child’s method of feeding (breastfeeding or through artificial mixtures) and, indirectly, the type of birth (natural delivery, caesarean section). It is estimated that the human gut microbiota is formed by approximately 2 years of age and undergoes continuous transformation over the next 3–5 years. However, there is no doubt that lifestyle—including nutrition, physical activity, as well as exposure to stressors—has a direct impact on the diversity of the human gut microbiota [3,7,8,9].

The composition of the adult microbiota is based on three types of bacteria, which include Firmicutes (*Lachnospiraceae*, *Ruminococcaceae*), Bacteroidetes (*Bacteroidaceae*, *Prevotellaceae*, *Rikenellaceae*), and Actinobacteria (*Bifidobacteriaceae*, *Coriobacteriaceae*). Due to the unique composition of the microorganisms residing in the gastrointestinal tract of everyone, three enterotypes are distinguished, which directly reflect the predominant bacterial assemblages in the intestine (*Bacteroidetes*—enterotype I, *Prevotella*—enterotype II, *Ruminococcus*—enterotype III) [9,10,11]. It seems worth noting that due to the complexity of the human gut microbiota, there are also bacteria that may show negative correlations depending on the enterotype in question. For example, for enterotype I, taxa such as *Methanobrevibacter* are distinguished; for enterotype II, *Akkermansia*; and for enterotype III, *Prevotella* (Table 1). The first two enterotypes are characterized by the dominance of bacteria from the genus Bacteroidetes, thus showing more diverse profiles compared to enterotype III, where the dominance of *Ruminococcus* appears to be less pronounced [11]. The current study indicates that enterotype is closely associated with a dietary pattern characterized by a high proportion of zoonotic products (mainly meat) in the diet. In contrast, enterotype II positively correlates with a high-carbohydrate diet based largely on products that are sources of simple sugars [9].

### 3.1. The Gut Microbiota in the Context of Cardiovascular Disease

Cardiovascular diseases (CVDs) represent a large global epidemiological burden and are, thus, the leading cause of death. Cardiovascular diseases (CVDs) include hypertension, cardiomyopathies, and ischemic heart disease, among others. Atherosclerosis is a component of CVD, where, because of obstructed blood flow in the blood vessels, dangerous associated complications occur. This process is the immediate stage leading to myocardial infarction, known as coronary artery disease, which develops slowly because of prolonged and cumulative exposure to a variety of risk factors, which include aspects of dietary behavior, genetic conditions, and even hormonal balance [1,2]. During atherosclerosis, patients are often co-morbid with other disease entities, where particular attention is paid to obesity and its complex pathomechanisms in relation to lipid metabolism disorders. Research to date clearly demonstrates the strong affinity between excessive body weight and impaired glucose tolerance, which in further stages contributes directly to type 2 diabetes (T2D). In turn, T2D has been recognized by the World Health Organization (WHO) as a major factor in cardiovascular complications [12,13]. There is no doubt, therefore, that current public health priorities are seeking solutions to introduce effective therapeutic interventions that contribute to a reduction in CVD mortality. Ongoing research clearly highlights the role of gut microbiota in aspects of CVC, thus opening up new space for understanding the relationship between the microbial environment in the gut lumen and a variety of cardiovascular health issues.

#### 3.1.1. Hypertension and the Gut Microbiota

Hypertension is a key modifiable risk factor for CVD. In terms of elevated blood pressure values, genetic factors play a relatively low contribution (<5%) compared to lifestyle. Research clearly emphasizes that reducing salt in the diet as well as normalizing body weight contributes to lowering blood pressure values. In addition, there are recommended dietary interventions, such as the Mediterranean diet or the DASH (*Dietary Approaches to Stop Hypertension*) diet, which have been shown to have hypotensive effects. Salt is also important for the intestinal microbiota, as it affects its composition, thereby inducing changes in its function. A high salt content in the diet can lead to a reduction in the diversity of the microbiota, particularly reducing the number of beneficial bacteria such as *Lactobacillus*. Studies carried out on the gut microbiota of people diagnosed with hypertension also showed that respondents had a higher abundance of Gram-negative bacteria, such as *Klebsiella*, *Parabacteroides*, *Desulfovibrio*, and *Prevotella*. It is noteworthy that Gram-negative bacteria are a source of pro-inflammatory endotoxins—lipopolysaccharides (LPS) [13,14].

Intestinal dysbiosis is defined as any change in the composition of microorganisms residing in the gut [3]. Previous scientific studies have shown that people with hypertension exhibit lower amounts of *Ruminococcaceae*, *Roseburia*, and *Faecalibacterium* spp., which are responsible for the production of short-chain fatty acids (SCFAs). SCFAs include propionic acid, acetic acid—used by the liver—and butyrate—used by colonocytes. Low SCFA levels can potentially contribute to the development of various metabolic syndromes, including HTN. The composition of the gut microbiota of HTN patients shows a lower diversity of *Butyricimonas* and *Corynebacterium*, which are responsible for the synthesis of sodium butyrate in the intestinal lumen. This metabolite contributes to reducing inflammation, modulating neurological and metabolic pathways. An important role for butyrate is also seen in processes related to the maintenance of intestinal epithelial integrity. Available scientific studies indicate that SCFAs may effectively contribute to lowering blood RR through a G-protein-related mechanism [2,13,14].

#### 3.1.2. Coronary Artery Atherosclerosis and the Gut Microbiota

Atherosclerosis is the most common cause of cardiovascular disease, and the process itself is the result of several risk factors, which include hypertension, lipid profile disorders, platelet hyperactivity, smoking, obesity, as well as diabetes. In addition, atherosclerosis is directly linked to chronic inflammation taking place in the human body. The resulting narrowing of the lumen of vessels (e.g., coronary arteries) can consequently lead to myocardial infarction [15,16].

The human gut microbiota has many important functions in the body; however, in terms of coronary artery atherosclerosis (CAD), lipid metabolism appears to be a key process. It mainly refers to the biosynthesis and degradation of lipids, i.e., fatty acids, triglycerides, and cholesterol. Lipoproteins are responsible for the transport of lipids to the liver, where fat metabolism processes occur. Fatty acids, on the other hand, have an antimicrobial role in the human body [15,17]. Research highlights that the composition of microbiota may correlate with atherosclerosis. Potential factors include the endotoxin LPS, which can enter the bloodstream through a damaged intestinal barrier and promote the development of atherosclerosis. In addition, LPS activates Toll-like receptor (TLR) signaling, which is responsible for immunity, thereby affecting levels of circulating cytokines such as tumor necrosis factor (TNF)-α [15].

**Figure 2:**
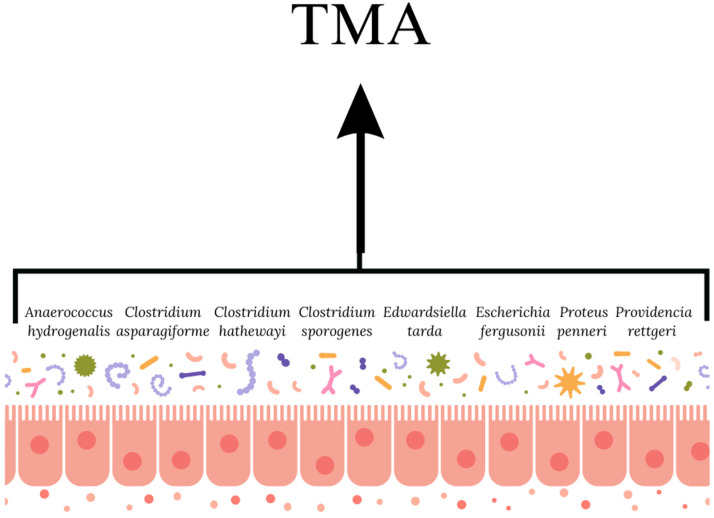
Bacterial strains involved in TMA synthesis (own elaboration based on [22,23]).

In relation to cardiovascular disease, much attention is focused on mechanisms related to the gut microbiota—primarily trimethylamine *N*-oxide (TMAO). Bacteria inhabiting the gut break down compounds such as carnitine, choline, or betaine into trimethylamine (TMA), which passes into the portal circulation and is then metabolized to TMAO by flavin monooxygenase (FMO) in the liver [18,19]. Research indicates that TMAO is crucial in the development of coronary atherosclerosis, which is also influenced by aspects such as cholesterol metabolism, short-chain fatty acids, and tryptophan metabolites [19,20]. The gut microbiota and serum metabolites are closely linked to the severity of ischemic heart disease, and bacteria such as *Roseburia*, *Ruminococcaceae,* and *Clostridium* can regulate the metabolic activity of bile acids (BA) and aromatic compounds, which affects the progression of coronary atherosclerosis. Numerous scientific studies confirm the correlation between elevated plasma TMAO levels and mortality and coronary incidents [15,19,21,22]. It seems noteworthy that TMAO levels increase with age and are excreted in the urine. Renal function is markedly better in young people compared to the elderly [22], so it seems reasonable to take care of the quality of the diet to reduce the amount of TMAO synthesized as much as possible (Figure 2).

In coronary artery disease (CAD), the gut microbiota is constantly being transformed. Research suggests that these changes are directly related to the severity of CAD. In a study by Liu et al. [24], it was shown that the numbers of bacteria from the *Lachnospiraceae* and *Ruminococcaceae* families were significantly lower compared to a healthy control group, and their total abundance decreased progressively with the development of CAD. Furthermore, *Ruminococcaceae* bacteria are inversely correlated with pulse wave velocity (PWV), an indicator of arterial stiffness [24,25]. Patients with coronary atherosclerosis also show a reduction in the number of *Bacteroides* and *Prevotella* and a higher quantitative proportion of *Streptococcus* and *Escherichia* [26,27].

### 3.2. Gut Microbiota in Cognitive Impairment

Increasing attention is focusing on the relationship between the composition of the gut microbiota and mental health. The available scientific literature highlights the role of the gut–brain axis in terms of cognitive impairment. The gut microbiome plays a key role in microglia function, influencing host immune and neuronal responses. An imbalance of the gut microbiota (dysbiosis) can lead to increased permeability of the intestinal epithelial barrier, resulting in the release of pro-inflammatory cytokines and promoting responses of the same nature [28].

Microbes show the ability to synthesize neurotransmitters (e.g., glutamate, GABA, serotonin, dopamine), and these, in turn, can indirectly participate in neuronal activity as well as host cognitive functions. It is noteworthy that the neurotransmitters do not cross the blood–brain barrier; nevertheless, neuropod cells, a subset of endocrine cells, can form synapses with vagus nerve neurons and release neurotransmitters (including glutamate) into the vagus nerve [29]. Breach of the blood–brain barrier (BBB) can be triggered by chronic inflammatory conditions, which include intestinal dysbiosis. Some researchers suggest that BBB disruption may be an important biomarker for the progression of diseases and conditions, including cognitive impairment [30]. However, there is a need for further research to confirm the above. An equally important component of the gut–brain axis is the hypothalamic–pituitary–adrenal axis (HPA), which is involved in the synthesis of stress hormones. Cortisol plays an important role in the development and function of the nervous system and in cognitive processes such as memory and learning [31]. The entire process involved in the stress response culminates in the release of glucocorticoids into the systemic circulation. These hormones, in turn, have the capacity to permeate the BBB, and prolonged exposure to stressors, both environmentally and endogenously, generates their release, contributing to the development of cognitive as well as cardiovascular dysfunction [32]. Abnormalities in HPA axis function are observed in depressed individuals, with the main factors being excessive glucocorticoid secretion and increased adrenocorticotropic hormone (ACTH) expression [32].

In terms of mood disorders, cognitive function, as well as appetite control and sleep, serotonin plays an important role. Commonly used drugs for mood disorders are SSRIs, which work by inhibiting serotonin reuptake at neuronal synapses in the brain. They block serotonin transporters (SERTs), leading to an increase in the concentration of this neurotransmitter in the synaptic space. Higher levels of serotonin improve the transmission of signals between neurons, which may improve mood and reduce symptoms of depression [33,34]. Tryptophan (an essential amino acid) is a precursor of serotonin, whose production in the brain is strongly linked to the gut microbiota. Gut bacteria can affect tryptophan metabolism, which, in turn, affects serotonin levels, thereby influencing mood and cognitive function [33,35]. Numerous studies in rodents (mice/rats) demonstrate links between the composition of the gut microbiota and depressive episodes. Bacterial strains such as *L. lactis* WHH2078 and *L. rhamnosus* IMC 501 are indirectly involved in tryptophan metabolism, contributing to a reduction in depressive-like disorders [36]. Gut microbiota-free (GF) mice had lower gut tryptamine levels and higher blood tryptophan levels than normal mice. This indicates that the gut microbiota mediates the conversion of tryptophan to tryptamine in the gut. The microorganisms involved in the above process include bacteria belonging to *Clostridium*, *Ruminococcus,* and *Lactobacillus* [35].

Cognitive dysfunction may be linked to abnormalities in dopaminergic signaling, which plays a key role in the regulation of mood, motivation, and thought processes. Short-chain fatty acids (SCFAs), produced by the gut microbiota during fiber fermentation, can influence brain function, thereby supporting neuronal function and modulating inflammation. Research indicates that some bacteria can synthesize or metabolize dopamine [33]. Thus, a study by Zhang et al. demonstrated that oral administration of berberine in mice contributed to increased dopamine production by gut bacteria [37]. In contrast, SCFAs, such as propionate, butyrate, and acetate, are mainly produced by bacterial fermentation, and recent studies have shown that SCFAs may play a role in gut–brain communication through the BBB, promoting its integrity. BBB leakage is observed in various neurodegenerative diseases, including stroke, epilepsy, multiple sclerosis, and Alzheimer’s disease [33,38].

## 4. Gut Microbiota of Patients with Coronary Artery Atherosclerosis and Cognitive Impairment

Multimorbidity is a common phenomenon in the elderly population over 65 years of age. Research has shown that patients with coronary atherosclerosis have a higher risk of developing cognitive impairment and dementia, which consequently correlates with a reduced quality of life for this group of patients, but also a significantly higher likelihood of serious cardiovascular incidents [39,40]. CAD, as well as cognitive dysfunction, is associated with changes in the composition of the gut microbiota, and, therefore, potential indicators taking on the role of biomarkers that would enable the planning of effective therapeutic interventions are seen in this area [40].

Dysbiosis, an imbalance of the gut microbiota, can lead to increased intestinal permeability, known as “leaky gut”. In this condition, the intestinal barrier loses its integrity, allowing toxins such as LPS to enter the bloodstream [41], and dysbiosis can contribute to the development of CAD as well as cognitive impairment [1]. A study by Yang et al. [42] found that a decrease in *Ruminococcaceae* bacteria and an increase in *Megamonas* were associated with cognitive impairment. The former group of bacteria was associated with reduced attentiveness and delayed recall, while the latter was also associated with delayed recall as well as impaired orientation. In Yang’s study, the hypertensive group had an increased abundance of *Megamonas*. In contrast, a study by Liu et al. [24] showed that the number of *Ruminococcaceae* decreased with the severity of CAD. On the other hand, a study by Toya et al. [43] indicates a reduced abundance of *Ruminococcus* gauvreauii in CAD patients. In addition, the authors of this paper highlight the role of the above taxon in terms of the synthesis of SCFAs—mainly acetate—which has been shown to have a protective effect in relation to hypertension. Both CAD and cognitive impairment adopt a common denominator in terms of risk factors, which include smoking, lipid disorders (mainly low-density lipoprotein cholesterol levels), hypertension, obesity, or hyperglycaemia. In view of the above, it is important to bear in mind that dysbiosis is also associated with the above-mentioned conditions, so establishing a causal relationship in terms of CAD and cognitive impairment can be problematic in many respects [44].

Not insignificant in terms of cognitive impairment is TMAO, which is a key element in the development of CAD. The above metabolite activates the transcription factor NF-κB, which, in turn, is an element that stimulates the synthesis of pro-inflammatory cytokines. TMAO-induced changes have been documented in hippocampal neurons, which can be attributed to modifications of synapses and neuronal plasticity. TMAO also promotes inflammation and oxidative stress, with excessive inflammatory response, as in atherosclerosis, being a key factor in the progression of Alzheimer’s disease (AD) [45]. A study by Vogt et al. [46] indicates that TMAO levels in the cerebrospinal fluid are elevated in people with dementia. In turn, elevated levels of the metabolite described by the authors in the cerebrospinal fluid are associated with AD severity, as well as neuronal degeneration. In contrast, a study by Gong et al. [47] suggested that TMAO could potentially be a marker of cognitive impairment, especially in post-stroke patients. The above study showed that post-stroke patients with cognitive impairment had higher serum TMAO levels compared to patients without impairment. However, it should be borne in mind that the mechanisms involved in the effects of TMAO in terms of cognitive decline require further study due to their complex nature [48].

## 5. Modulation of the Gut Microbiota in Relation to CAD

Proper nutrition is an important part of CVD prevention. There is also no doubt that diet has a direct impact on the diversity of the gut microbiota, thus being a modifiable factor of it [2,3,9,27,49]. Numerous scientific studies have demonstrated that the composition of the gut microbiota in patients with CAD differs from that of healthy subjects [15,18,27]. Interventions aimed at restoring an appropriate balance of microorganisms in the gut, such as a fiber-rich diet and probiotics, may prove helpful in inhibiting the progression of atherosclerosis by modulating the production of harmful metabolites—such as TMAO—and reducing inflammation. In addition, research into specific bacterial strains offers new therapeutic options for both the prevention and treatment of atherosclerosis [50].

### 5.1. Dietary Fiber

Dietary fiber plays a key role in modulating the gut microbiota, which is crucial in the prevention and treatment of atherosclerosis. Fermentation of fiber by gut bacteria results in the production of SCFAs, including butyrate, propionate, and acetate, which, in turn, exhibit anti-inflammatory properties. In addition, they may increase the integrity of the intestinal barrier, thereby reducing the translocation of pro-inflammatory LPS into the bloodstream [15,50,51].

Enterolignans, mainly enterolactone and enterodiol, are substances produced by intestinal bacteria from plant lignans, which are found in products that are sources of dietary fiber, such as whole-grain cereal products, pulses, seeds such as flax, vegetables, and fruit. The presence of high levels of enterolactone has been linked to a reduction in TMAO levels, which is associated with a fiber-rich diet. A cohort study by Liu et al. [52] found that the above ratio of enterolactone to TMAO levels was associated with a lower risk of developing CAD. Similar results were obtained by Hu et al. [53], who showed that long-term lignan intake was associated with a lower risk of coronary artery disease in both genders.

TMAO is a metabolite produced by gut microbiota from choline and L-carnitine, which are abundant in red meat and other animal products [18,19]. Vegetarian diets have been linked to a reduction in TMA production by microbiota. In contrast, diets high in oligosaccharides stimulate an increase in *Prevotella* bacteria, which, in turn, reduces the availability of choline for TMA synthesis [54]. Bacterial strains of *Turicibacter* and *Bifidobacterium* involved in cholesterol metabolism are associated with lower plasma levels of TMA and TMAO, and the increase in commensal bacteria in one study increased with additional supplementation of fermented dietary fiber [55,56]. Dietary models based on adequate amounts of dietary fiber may contribute to the reduction in TMA precursors to TMAO, resulting in a potential effective therapeutic intervention to reduce TMAO levels by modulating the composition of the gut microbiota [57]. However, there is a need for further research in this direction to understand the cause-and-effect relationship in terms of the association between modulation of gut microbiota and CAD.

### 5.2. Probiotics

Probiotics may play an important role in the prevention and treatment of coronary atherosclerosis via the gut microbiota. It appears that *L. plantarum* shows beneficial effects in terms of TMAO, lowering its serum levels. Furthermore, the above strain may contribute to the alleviation of cognitive impairment, which is becoming a common problem in patients with coronary atherosclerosis [39,40,50]. *Lactobacillus rhamnosus GG* appears to be the most effective strain showing plasma TMAO-lowering effects, as confirmed by studies in mouse models as well as clinical trials to date [58]. In contrast, a meta-analysis in 2023 [59] shows that probiotic supplementation has no significant effect on lowering plasma TMAO concentrations. However, it should be borne in mind, as also emphasized by the authors, that different bacterial strains were included, which makes it impossible to draw a definite conclusion.

## 6. Summary

Previous research suggests an important role for gut microbiota in coronary artery atherosclerosis and cognitive impairment. An imbalance of the microbiota, known as dysbiosis, can lead to increased production of harmful metabolites. Currently, one of the most important metabolites of gut microbiota is TMAO, which promotes inflammation and the deposition of atherosclerotic plaques. Dysbiosis can affect gut permeability, allowing toxins to enter the bloodstream, which causes chronic inflammation and negatively affects cognitive function. The above article may also highlight potential therapeutic interventions, such as dietary modifications or the use of probiotics, which may improve mental health and cognitive function in patients with coronary atherosclerosis, offering new approaches to the comprehensive management of this disease. Nevertheless, further research, particularly clinical research, is needed to gain a deeper understanding of the mechanisms of action of gut microbiota and its metabolites, as well as their impact on the development of atherosclerosis and cognitive impairment.

## Figures and Tables

**Table 1:** Characteristics of enterotypes—own elaboration based on [9,11].

	Enterotype I	Enterotype II	Enterotype III
Predominant bacterial type	*Bacteroidetes*	*Prevotella*	*Ruminococcus*
Type of bacteria	*Parabacteroides*, *Alistipes*, *Bilophila*	*Desulfovibrio*, *Succinivibrio*.	*Ruminoccocus* *Akkermansia* *, *Methanobrevibacter* *
Type of bacteria potentially negatively associated (e.g., with inflammatory processes or dysbiosis)	*Methanobrevibacter*	*Akkermansia*	*Prevotella*

* Co-occurring taxa—bacteria that frequently occur alongside the dominant genus in a given enterotype. Although not numerically dominant, they may play an important role in regulating the metabolic and immune functions of the gut microbiota.

## Abbreviations

The following abbreviations are used in this manuscript:

ACTH	Adrenocorticotropic hormone
AD	Alzheimer’s disease
BBB	Blood–brain barrier
CAD	Coronary artery atherosclerosis
CVD	Cardiovascular diseases
DASH	Dietary Approaches to Stop Hypertension
HPA	Hypothalamic–pituitary–adrenal axis
LPS	Lipopolysaccharide
SCFAs	Short-chain fatty acids
TMAO	Trimethylamine *N*-oxide
